# Investigation and Tailoring of Rotating Squares’ and Rectangles’ Auxetic Structure Behavior through Computational Simulations of 6082T6 Aluminum Alloy Structures

**DOI:** 10.3390/ma16247597

**Published:** 2023-12-11

**Authors:** Mahmoud Elsamanty, Hassan Elshokrofy, Abdelkader Ibrahim, Antti Järvenpää, Mahmoud Khedr

**Affiliations:** 1 Mechanical Engineering Department, Faculty of Engineering at Shoubra, Benha University, Cairo 11629, Egypt; hassan.selim@feng.bu.edu.eg (H.E.); abdelkader.ibrahim@feng.bu.edu.eg (A.I.); 2 Mechatronics and Robotics Department, School of Innovative Engineering Design, Egypt Japan University of Science and Technology (E-JUST), Alexandria 21934, Egypt; 3 Kerttu Saalasti Institute, Future Manufacturing Technologies (FMT), University of Oulu, 85500 Nivala, Finland; antti.jarvenpaa@oulu.fi

**Keywords:** auxetic structures, re-entrant structure, rotating rectangles, rotating squares, finite-element analysis

## Abstract

Auxetic structures, renowned for their unique lateral expansion under longitudinal strain, have attracted significant research interest due to their extraordinary mechanical characteristics, such as enhanced toughness and shear resistance. This study provides a systematic exploration of these structures, constructed from rigid rotating square or rectangular unit cells. Incremental alterations were applied to key geometrical parameters, including the angle (θ) between connected units, the side length (*a*), the side width (*b*) of the rotating rigid unit, and the overlap distance (*t*). This resulted in a broad tunable range of negative Poisson’s ratio values from −0.43 to −1.78. Through comprehensive three-dimensional finite-element analyses, the intricate relationships between the geometric variables and the resulting bulk Poisson’s ratio of the modeled auxetic structure were elucidated. This analysis affirmed the auxetic behavior of all investigated samples, characterized by lateral expansion under tensile force. The study also revealed potential stress concentration points at interconnections between rotating units, which could impact the material’s performance under high load conditions. A detailed investigation of various geometrical parameters yielded fifty unique samples, enabling in-depth observation of the impacts of geometric modifications on the overall behavior of the structures. Notably, an increase in the side width significantly enhanced the Poisson’s ratio, while an increase in the overlap distance notably reduced it. The greatest observable change in the Poisson’s ratio was a remarkable 202.8%, emphasizing the profound influence of geometric parameter manipulation. A cascaded forward propagation–backpropagation neural network model was deployed to determine the Poisson’s ratio for auxetic structures, based on the geometric parameters and material properties of the structure. The model’s architecture consisted of five layers with varying numbers of neurons. The model’s validity was affirmed by comparing its predictions with FEA simulations, with the maximum error observed in the predicted Poisson’s ratio being 8.62%.

## 1. Introduction

Auxetic materials, characterized by a negative Poisson’s ratio, have garnered significant attention within the scientific community due to their mechanical behaviors, which defy conventional expectations. Unlike typical materials that contract under tensile stress, auxetic specimens consistently exhibit lateral expansion [1,2]. Exceptional attributes, including increased toughness, improved shear resistance, and superior resistance to indentation, further accentuate this unique mechanical response. These distinctive qualities have significantly broadened the applicative scope of auxetic materials, fostering transformative breakthroughs across diverse disciplines. The anomalous behavior of auxetic materials has profound implications for applications in various fields, such as biomedical engineering and safety equipment. In biomedical engineering, the distinctive characteristics of auxetic materials offer unprecedented opportunities for revolutionizing device design. Meanwhile, in safety equipment, auxetic materials contribute to substantial advances in impact mitigation by providing superior vibration damping and shock absorption capabilities [1]. This versatility underscores the potential of auxetic materials as essential components in the advancement of various scientific and technological domains.

Moving beyond their intriguing mechanical properties, the exploration of auxetic materials has been a focal point in the field of material science. Early studies have revealed a crucial link between the auxetic behavior of these materials and their geometric properties, sparking a surge in research dedicated to engineering auxetic structures through the meticulous design and manipulation of geometric features [3]. Investigations encompass both natural and synthetic auxetic materials, resulting in various fabrication techniques for crafting adjustable auxetic structures. Despite significant progress, certain aspects of the intricate relationships between geometric parameters and the resulting mechanical behavior remain incompletely understood [3]. Existing research focuses mainly on a limited range of geometrical parameters to analyze their impact on the Poisson’s ratio values [4]. Rather than diminishing the strides achieved in the field, this underscores specific areas where further exploration could yield valuable insights and advancements.

Building on this foundation, a noteworthy breakthrough has unfolded in auxetic materials’ research, driven by the integration of advanced manufacturing techniques. These technological advances have substantially augmented the ability to craft inventive auxetic geometries and fine-tune their mechanical attributes. Consequently, a new and promising category of auxetic metamaterials, known as auxetic lattice structures formed by rotating rigid units, has emerged [5,6,7]. However, despite these advancements, the existing literature provides only a limited comprehension of how crucial geometric variables impact the Poisson’s ratio within these structures. Of particular interest is the remarkable ability of auxetic materials to undergo lateral swelling under tensile forces and contract when compressed [8]. Furthermore, these materials exhibit superior mechanical properties, including increased shear stiffness [9], increased fracture toughness [10], increased resistance to indentation [11], and a superior capacity for energy dissipation [12,13,14]. However, despite these promising attributes, the exploration of these characteristics remains an ongoing endeavor, and certain aspects of the subject are still awaiting a comprehensive exploration.

Auxetic materials present a myriad of potential applications that transcend a diverse range of disciplines [15]. One such application is found in energy and shock absorption systems, wherein auxetic lattice structures have emerged as potential Pioneers. Their unique ability to expand under tension and contract under compression makes them ideal candidates for effective energy dissipation and impact absorption, thus enhancing the durability and performance of such systems [16,17]. In the realm of biomedical engineering, auxetic materials possess significant potential to revolutionize the design and functionality of implants and prosthetics [18]. Their distinctive mechanical properties, such as increased toughness and resistance to indentation, coupled with the ability to tailor their geometric properties, could improve the performance, longevity, and biocompatibility of these devices. It could lead to improved patient outcomes, offering a higher quality of life for individuals reliant on these medical aids. Moving to the sports industry, the self-protective qualities of auxetic materials could transform the landscape of protective gear for athletes [19]. The unique ability of these materials to dissipate energy and withstand impact could provide superior protection, reduce the risk of injuries, and improve performance. Moreover, the flexibility of the design offered by auxetic materials could allow the creation of more-comfortable and -ergonomic equipment, further enhancing its utility in this field.

Delving deeper into the structural aspects of these fascinating materials, the quest for novel auxetic materials often necessitates the identification and manipulation of specific geometric attributes that induce auxetic behavior [20,21]. This endeavor has led to the delineation of three fundamental types of auxetic structures, each distinct in its deformation mechanisms and corresponding geometries. These include re-entrant structures, which are available in both 2D and 3D configurations, offering versatility in application [16,22,23]. Chiral structures represent another category, showing unique mechanical properties and design potential [24,25,26,27,28]. However, it is the rotating rigid unit structures that have garnered significant attention as a promising class of lightweight auxetic materials [22,29,30,31]. Their intriguing mechanical properties, coupled with design flexibility, offer a promising avenue for exploration and application. These structures, characterized by units that rotate under stress, offer the potential to create materials with customized responses to external forces, thus expanding the horizons of auxetic material applications [4,32,33,34,35,36]. In summary, the inherent versatility and adaptability of auxetic materials promise a future where the materials’ properties are not just a function of their composition, but are intricately tied to their geometric design, opening a new frontier in materials science and engineering.

As we pivot our focus from the structural foundations of auxetic materials to their real-world applications, it becomes clear that the expansive benefits of these materials are Tangible, yet their actualization hinges significantly on a comprehensive understanding of the geometric parameters that dictate their unique behaviors [37,38,39]. The relentless pursuit of this comprehension forms the crux of progressive advancements in auxetic materials, paving the way for their incorporation into a diverse range of applications. These encompass innovative medical devices that promise improved fit coupled with superior comfort, protective gear that delivers exceptional energy absorption and dissipation, and the development of electronic devices that can withstand mechanical stress with resilience while preserving flexibility [40,41,42,43].

Building on this understanding, the focal point of our investigation is to deepen our collective knowledge of auxetic behavior by meticulously examining the association between the geometric parameters and the Poisson’s ratio exhibited in auxetic structures. The insights gleaned could potentially serve as a valuable blueprint for the design and creation of auxetic materials, thus paving the way for the emergence of novel materials characterized by customized mechanical properties [44,45,46]. Consequently, this study is anticipated to contribute significantly to the overarching goal of engineering future materials. It is envisioned not only through the modification of the chemical composition, but also through the strategic tailoring of the geometrical parameters to actualize unusual and highly beneficial mechanical properties. This approach to material design is a transformative paradigm shift, blending the principles of chemistry, physics, and engineering to harness the potential of auxetic materials truly. As such, the study hopes to add a new dimension to materials science by exploiting the possibilities inherent in their geometric configurations [47].

Looking forward, the ambition is to propel the field of materials science into a new era where geometric manipulation becomes a potent tool in the creation of materials with properties tailored to specific applications. Despite the significant advancements in the study of auxetic materials and the recognition of their potential in various industries, several gaps in knowledge remain. The relationship between the geometric parameters and the Poisson’s ratio in these materials, particularly in auxetic structures formed by rotating rigid units, has not been fully explored nor understood [48]. The work proposed in [49] represents a crucial gap in our understanding, given that this relationship plays a key role in determining the overall mechanical behavior of auxetic materials. Additionally, while the unique mechanical properties of auxetic materials have been recognized, the specific impact of these properties in practical applications, such as in biomedical devices or shock-absorption systems, is not well understood. More research is needed to understand how the mechanical characteristics of auxetic materials can be optimized for specific applications [50]. Therefore, the final stage of this study aimed to address these gaps by focusing on the investigation of the relationship between the geometric parameters and Poisson’s ratio in auxetic structures and by examining how these relationships can be utilized to optimize auxetic materials for various applications [51].

This research is anchored by a multifaceted objective, central to which is the exploration of the behavior and potential of auxetic structures, particularly those composed of rotating rigid square and rectangular units. A systematic investigation of these structures was conducted, utilizing computational modeling as the principal analytic tool. The secondary objective involved an exhaustive analysis of the impact of key geometric variables on the Poisson’s ratio. It is anticipated that this deep dive could yield structured design guidelines, which could subsequently facilitate the adjustment and customization of auxetic structures to meet diverse application requirements. The ultimate aim lies in the potential engineering of auxetic materials with tailored properties, a goal that could be realized through the application of the design guidelines mentioned above. The research also encompasses an analysis of fifty unique geometries derived from systematic manipulations of four core geometric parameters: the angle (θ) between two connected units, the side length (*a*) and side width (*b*) of the rotating rigid unit, and the overlap distance (*t*), which functions as a spring hinge connecting the rotating rigid units within the structure. This comprehensive examination is expected to shed light on the influence of these geometric parameters on the Poisson’s ratio and the overall mechanical behavior of the auxetic structure. Ultimately, the research is poised not only to deepen the understanding of auxetic structures, but also to provide practical, actionable guidelines for their design and engineering, thus contributing to the broader field of materials science.

## 2. Methodology

### 2.1. Geometrical Parameters’ Determination

**Figure 1:**
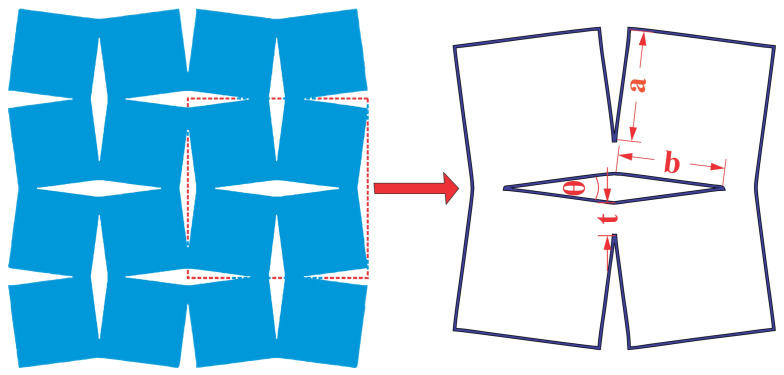
Geometric parameters for rotating square and rectangular auxetic structure selection.

The primary focus of this research lied in carefully selecting geometric parameters for the proposed auxetic structures, which play a pivotal role in determining their mechanical behavior. The chosen parameters, as illustrated in Figure 1, encompass several key aspects: the angle (θ) between the two connected units, denoted by (a,b), representing the side length and side width of the rotating rigid unit, and (*t*), which signifies the overlap distance acting as a spring hinge connecting the rotating rigid units within the structure. To comprehensively understand the behavior of the lattice structures across various configurations, a broad spectrum of values was systematically chosen for each parameter, as documented in Table 1. This extensive range of values allowed for an in-depth exploration of the structural response under different geometric settings, facilitating a comprehensive analysis of the structure’s mechanical properties.

After determining each sample’s dimensions, a computer-aided design (CAD) model was meticulously created for every structure. Advanced design software, SOLIDWORKS 2021, enabled the precise construction of the samples according to the specified dimensions. This CAD modeling process ensured that the samples were fabricated accurately and adhered to the desired geometric parameters. Subsequently, the output files generated from the CAD models were transformed into a suitable format for analysis employing FEA software. Finite-element analysis, a powerful computational technique, allowed for the simulation and evaluation of the mechanical behavior of the auxetic structures under various loading conditions. By subjecting the structures to virtual tests and assessments, FEA provided valuable insights into their deformation characteristics, stress distribution, and overall performance.

The integration of CAD modeling and FEA analysis in this research served as an indispensable framework for investigating the selected geometric parameters and their influence on the mechanical behavior of the auxetic lattice structures. Combining these advanced tools and techniques facilitated the design and fabrication process. It enabled a comprehensive understanding of the structural response, paving the way for informed decision-making and optimizing the auxetic structures. Overall, the rigorous methodology employed in this study, encompassing the selection of the geometric parameters, CAD modeling, and FEA analysis, ensures a systematic and scientific approach to investigating the behavior of auxetic lattice structures. This research framework provides a solid foundation for the subsequent analysis and interpretation of the results, ultimately advancing knowledge in auxetic materials and their applications.

### 2.2. Finite-Element Study of Structural Behavior

To comprehensively investigate the behavior of the aforementioned structure, a sophisticated three-dimensional finite-element model was developed and implemented. This analytical approach was employed as a powerful tool to delve into the mechanics and intricacies underlying the response of the structure under various conditions. The widely recognized ANSYS 2021 R2 software was utilized for this purpose, leveraging its advanced capabilities and robust simulation techniques. By employing the ANSYS software, the ability to meticulously examine and analyze the performance characteristics of the structure was achieved. This encompassed the study of the deformation patterns, stress distribution, and load-bearing capacity across different scenarios and loading conditions. The finite-element method within ANSYS enabled the accurate representation of the complex geometry and material properties of the structure, allowing for a realistic simulation of its mechanical behavior. By subjecting the finite-element model to virtual tests and simulations, valuable insights into the structural response and its sensitivity to different factors were obtained. This included evaluating the effects of various external loads, boundary conditions, and material properties on the structure’s performance. A thorough analysis of the simulation results enabled the identification of critical areas of stress concentration, the assessment of structural integrity, and informed decision-making regarding design modifications or optimizations. The adoption of the ANSYS software and the application of finite-element analysis in this study provided a rigorous and systematic framework for investigating the behavior of the structure. This approach not only enhances the understanding of the fundamental mechanics, but also facilitates the identification of potential design improvements and optimization strategies. The insights gained from the finite-element model analysis contribute to the advancement of knowledge in the field, enabling the development of more-efficient and -reliable structures for practical applications.

### 2.3. Optimization of Meshing

The initial step in the finite-element analysis (FEA) model was selecting the input material. The aluminum alloy designated as AA6082T6 was deliberately chosen for its appropriateness within the context of this research endeavor. Renowned for its medium-strength, heat-treatable characteristics, AA6082T6 possesses commendable corrosion resistance and weldability, making it a frequent choice in structural engineering applications. It is commonly denoted as AA6082T6 within industry discourse. In order to uphold the precision of our simulation outcomes, a comprehensive suite of material properties pertinent to this alloy was procured from standardized data housed in the software database. This set of material properties encompasses a range of essential characteristics crucial to our finite-element analysis (FEA) model, including, but not limited to the elastic modulus, Poisson’s ratio, density, yield strength, and thermal expansion coefficient. These parameters collectively contributed to the robustness of our FEA model, as succinctly outlined in Table 2. The selection of AA6082T6 as our material of choice emanated from its widespread utilization across industrial domains and its possession of properties deemed well-suited for the operational conditions encapsulated within our model. The deliberate inclusion of AA6082T6 in our study served the purpose of establishing a thorough and transparent foundation for our model. The alloy’s extensive prevalence in industrial applications, combined with its favorable attributes within the pertinent operational conditions, fortifies the reliability and reproducibility of our study. This intentional selection aimed to furnish our readers with a nuanced comprehension of the material selection rationale, thereby enhancing the overall integrity and scholarly merit of our research endeavor.

**Figure 2:**
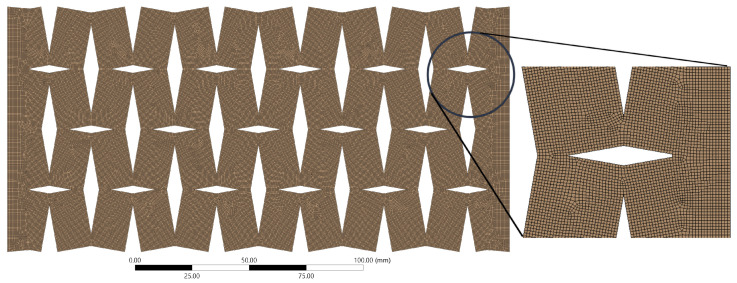
Illustration of the computational model depicting the detailed meshing of the auxetic structure.

In developing our model, we adopted a meticulous approach to select the meshing configuration. This process involved the use of the patch-confirming method and quadratic element order, with a consistent element size of 0.5 mm. This element size was not arbitrarily chosen, but was determined based on the convergence requirements of our solution, which were particularly sensitive to small values of *t* and θ. The final meshing configuration, illustrated in Figure 2, was the result of a careful process of optimization. It effectively balanced accuracy and computational efficiency, thereby providing a reliable representation of the structural behavior, even when dealing with smaller values of *t* and θ.

**Figure 3:**
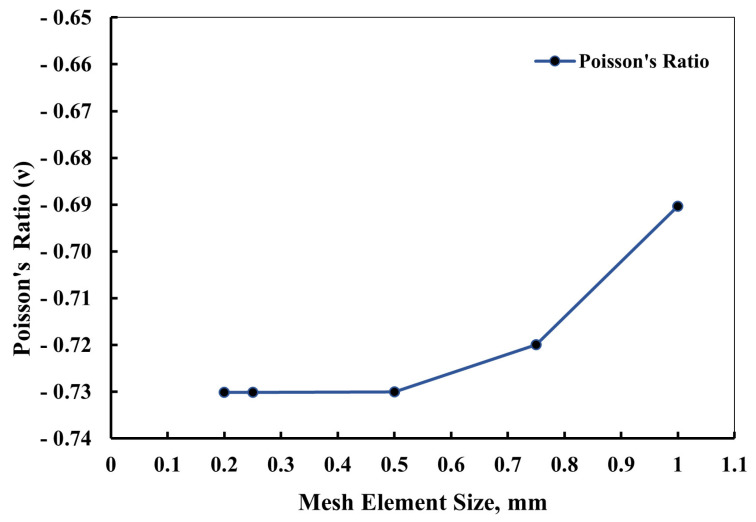
Illustration depicting the careful selection process of the meshing element size, crucial for accurate finite-element analysis of the structural behavior. The chosen element size ensures an optimal balance between computational efficiency and solution accuracy, contributing to reliable simulations.

Figure 3 offers an exploration of the influence of varying element sizes on the Poisson’s ratio values, rather than a definitive argument for convergence. As the element size decreased from 0.5 to 0.25 and 0.2 mm, the magnitude of the Poisson’s ratio values reduced. However, beyond this point, the changes in these values became negligible, suggesting that an element size of 0.5 mm was sufficient for the solution process. This choice had the added benefit of reducing the computational costs and saving valuable time during the analysis.

### 2.4. Boundary Conditions and Constraints

**Figure 4:**
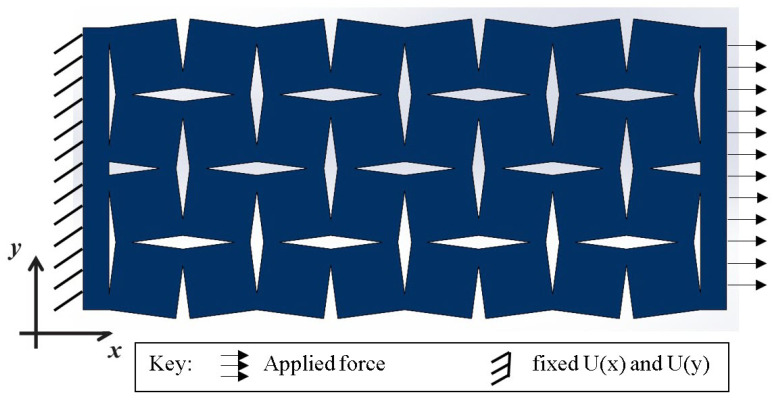
Boundary conditions for FEA model rotating squares’ and rotating rectangles’ auxetic structure.

In the computational model under examination, meticulously defined boundary constraints were implemented to simulate real-world conditions. As depicted in Figure 4, a fixed support was applied along one edge of the complete geometric representation, limiting movement along this boundary and creating a stable reference point for the analysis. This specific positioning of the fixed support provided a foundational framework upon which the subsequent forces and deformations could act. In contrast to the fixed edge, an axial force of 500 N was exerted in the X-axis direction on the opposite edge of the structure. This force application was designed to induce deformation primarily within the X-Y plane, which was the plane of interest for this study. The directional force applied mirrored the conditions the structure would likely encounter in its intended applications, providing valuable insights into its behavior under such circumstances. The magnitude of the force applied, set at 500 N, was not arbitrarily chosen. It was meticulously calibrated through preliminary analyses to ensure that the induced stress would retain the structure well within its elastic region, far below its yield point. This precautionary measure ensured that the structure maintained rigidity and did not enter plastic deformation during the investigation. The careful selection of this force value allowed for an accurate study of the elastic deformation characteristics of the auxetic structure, thus providing a reliable evaluation of its mechanical performance under controlled conditions.

From the results of the computational analysis, specific data points were selected for a detailed examination. These included the equivalent von Mises stress, which is a scalar value derived from the three-dimensional stress state in the structure, providing a comprehensive view of the stress distribution within the model. Equivalent strain was also analyzed, offering insights into the overall deformation that the structure experienced under the applied force. In addition to these global measures, localized deformations were also considered. Specifically, deformation along the X-axis ($\Delta L_{x}$) and Y-axis ($\Delta L_{y}$) was monitored. These values provided an understanding of how the structure reacted to the applied force in individual directions, thereby offering a more-granular perspective on the deformation characteristics of the model.

Furthermore, the Poisson’s ratio (ν)—a critical material property that defines the ratio of transverse strain to axial strain—was calculated using the formula:

(1) $$\nu =-\frac{\varepsilon_{y}}{\varepsilon_{x}}$$

where $\varepsilon_{y}$ and $\varepsilon_{x}$ are the lateral and longitudinal strain, respectively. The strains were calculated using the formulae $\varepsilon_{y}=\frac{\Delta L_{y}}{L_{y}}$ and $\varepsilon_{x}=\frac{\Delta L_{x}}{L_{x}}$, where $\Delta L_{y}$ and $\Delta L_{x}$ are the deformations in the Y and X directions, respectively, and $L_{y}$ and $L_{x}$ are the original lengths of the specimen in the respective directions. The original lengths $L_{y}$ and $L_{x}$ were predetermined from the CAD model of the structure, ensuring accuracy in the calculation of the strains. It is crucial to note that these lengths corresponded to the dimensions of the structure prior to any force application or deformation. Through these calculations and selections of the data, a comprehensive understanding of the material behavior and deformation characteristics under the applied load was achieved.

## 3. Results and Discussion

**Figure 5:**
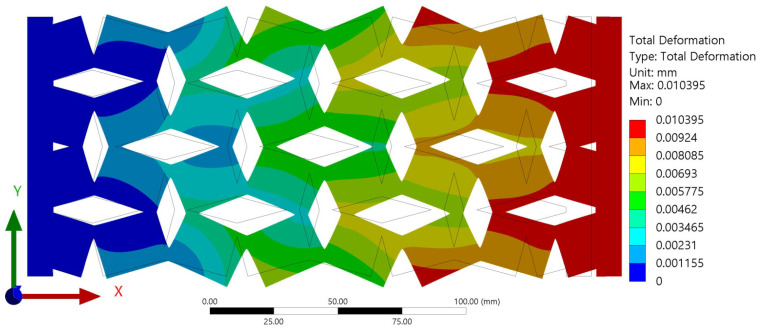
Auxetic behavior shown from deformation of the sample with a=15mm, b=20mm, θ=30°, and t=5mm.

**Figure 6:**
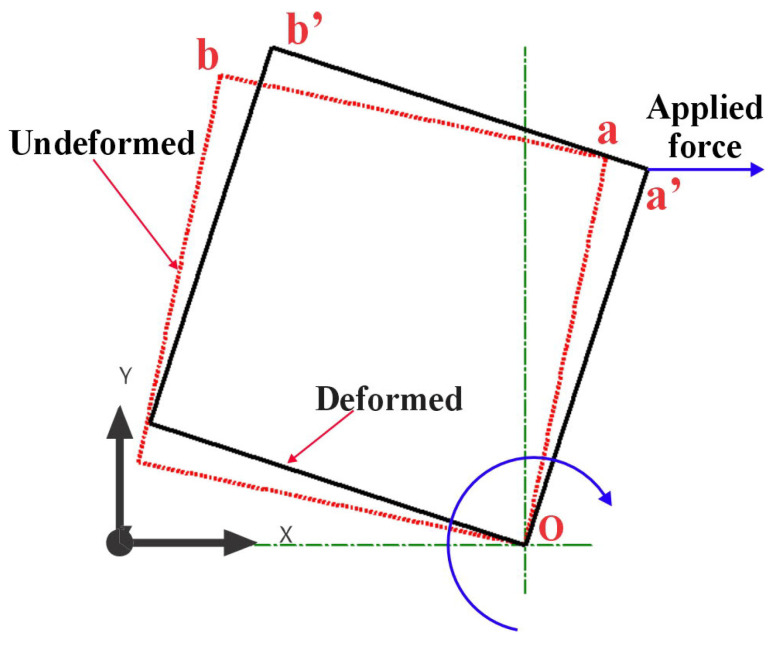
Rotation mechanism for single unit cell due to longitudinal force.

The finite-element analysis (FEA) results provided clear evidence that all investigated samples exhibited auxetic behavior, a characteristic of materials that exhibit an unusual mechanical response of expanding laterally when stretched. The auxetic nature of the samples was particularly noticeable, as demonstrated in Figure 5, which depicts the behavior of one sample under tensile force. This figure presents a comparison of the wireframe model of the sample both before and after deformation. The structural changes depicted in the wireframe model offer a visual representation of the auxetic behavior of the material under tensile forces. The auxetic behavior induced by the rotating squares and rectangles in the auxetic structure array is represented more comprehensively in Figure 6. The force at point (a), acting in the X-axis direction, instigated a rotational movement of the unit cell about point (O). This point (O) can be considered as a fixed pivot relative to the unit cell, serving as the center of rotation for the induced structural changes.

As the unit cell rotates under the influence of the applied force, points (a) and (b) are displaced to new positions denoted as (a′) and (b′), respectively. This displacement is indicative of the expansion of the structure in both the lateral and longitudinal directions, a clear manifestation of the auxetic behavior. It is worth noting that this simultaneous expansion in both directions is atypical of conventional materials, underscoring the unique mechanical properties of the studied auxetic structures. Moreover, the rotation of the unit cell and the concomitant deformation in response to the applied force provided insightful data into the internal mechanisms governing the auxetic behavior of the structure. Understanding these mechanisms is crucial for tailoring the design of the unit cell to achieve the desired mechanical properties and behavior in various applications.

**Figure 7:**
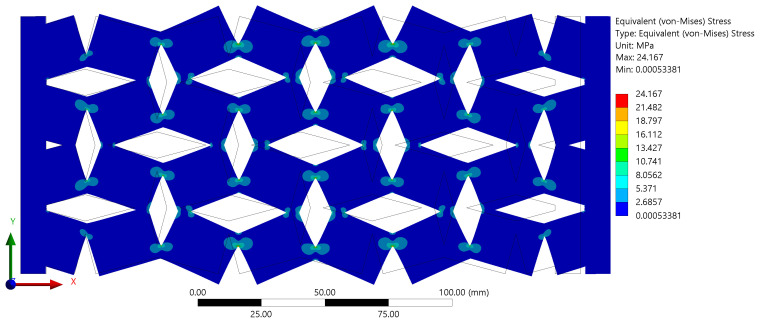
Stress values for the sample with a=15mm, b=20mm, θ=30°, and t=5mm.

The objective was to validate that the samples had not undergone any plastic deformation, which would indicate that the material has been stressed beyond its elastic limit and result in a permanent change in shape even after the removal of the applied force. This confirmation of elastic behavior is crucial as it ensures that the observed auxetic properties are inherent to the material structure and not a result of permanent, plastic deformation. The distribution of stress along the surface of the samples, as presented in Figure 7, was another area of keen interest. It was observed that the regions of interconnection between the rotating units exhibited the highest stress values.

This increase in stress can be attributed to the phenomenon of stress concentration, which is typically induced by geometric discontinuities such as sharp corners, notches, or variations in cross-sectional areas. In the case of our samples, the sharp corners at the interconnections between rotating units served as the points of stress concentration. This stress concentration at the interconnections can have significant implications for the overall mechanical behavior of the auxetic structure. It could potentially be a limiting factor in the material’s performance under high-load conditions.

**Figure 8:**
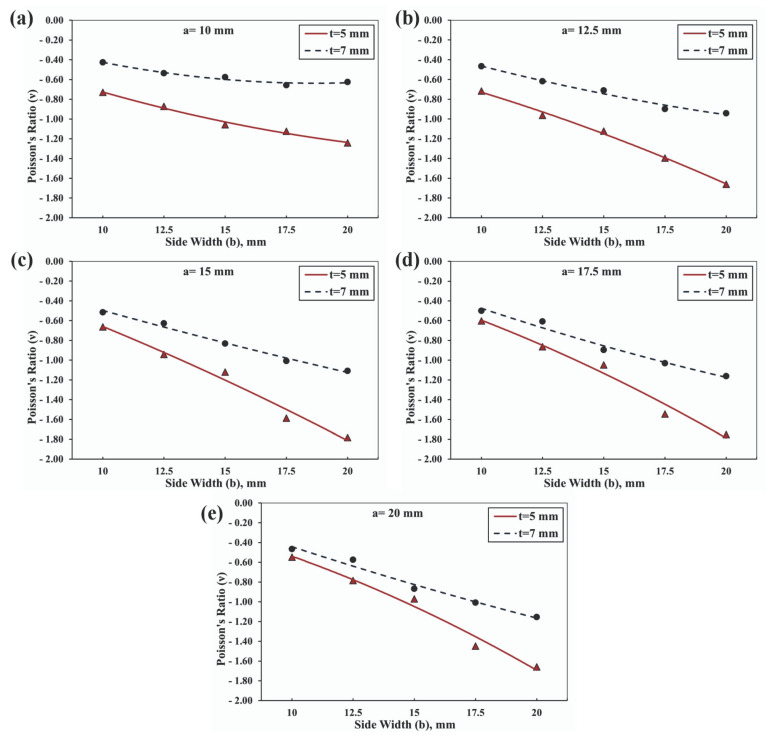
Evolution of the Poisson’s ratio values at different side widths and thicknesses for rotating squares and rectangles having θ=30°, at different side length values of: (**a**) 10 mm, (**b**) 12.5 mm, (**c**) 15 mm, (**d**) 17.5 mm, and (**e**) 20 mm.

The investigation into the influence of various geometrical parameters resulted in the generation of fifty unique samples, each distinguished by their inherent characteristics. This comprehensive study facilitated the observation of the impacts of the geometric alterations on the overall behavior of the structures, which are presented in Figure 7. Notably, it was observed that an enhancement in the side width, while keeping the side length constant at 10 mm, induced a significant increase in the Poisson’s ratio, as illustrated in Figure 8a. This pattern was consistently observed across all graphical representations, indicating a direct relationship between the side width value and the Poisson’s ratio of the investigated auxetic structures. Furthermore, the investigation extended to the role of the overlap distance in the manifestation of the auxetic properties of the structures.

It was observed that an increase in the overlap distance from 5 mm to 7 mm instigated a notable reduction in the Poisson’s ratio. This observation reinforced the substantial influence of the overlap distance on the auxetic behavior of the structures, suggesting that a meticulous manipulation of this parameter could yield the desired Poisson’s ratio values, thereby enabling the tuning of the material’s mechanical response. Upon examining the alterations in the Poisson’s ratio with respect to changes in the geometrical parameters, it was discerned that the greatest observable percentage change was 70.3%, as shown in Figure 8a. The subsequent figures revealed an even more-significant change; the highest observable percentage change in the Poisson’s ratio amount to 131.8% in Figure 8b, 168.9% in Figure 8c, 191.08% in Figure 8d, and a staggering 202.8% in Figure 8e. These dramatic changes underscored the profound impact that the manipulation of geometrical parameters can have on the Poisson’s ratio. Such findings pave the way for the precise tuning of auxetic materials’ mechanical responses, presenting significant implications for advancements in materials science and engineering.

**Figure 9:**
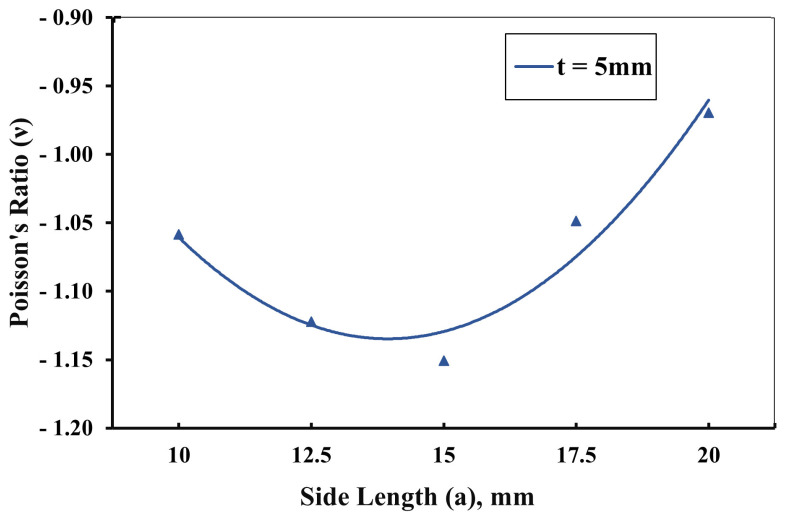
Effect of changing side length *a* on Poisson’s ratio at *b* = 15 mm and *t* = 5 mm.

A more-detailed exploration of the geometrical parameters was conducted on samples with a side width of 15 mm and an overlap distance of 5 mm. The data visualized in Figure 9 revealed a fascinating trend: an increase in the side length from 10 mm to approximately 15 mm led to an increased Poisson’s ratio. However, an intriguing reversal of this trend was observed when the side length exceeded 15 mm, which resulted in a decrease in the Poisson’s ratio. This observation remained consistent across all samples and revealed an interesting correlation: when the parameters were kept constant, an increase in the side length led to an augmentation of the Poisson’s ratio. This trend persisted until the moment the side length equaled the side width, transforming the geometry into a square. Contrary to expectations, at this point, the Poisson’s ratio began to decline. This phenomenon suggested that the square geometry of the structure introduced a unique dynamic in the auxetic behavior, meriting further exploration to fully understand its implications.

## 4. Poisson’s Ratio Estimation

The determination of the Poisson’s ratio for auxetic structures was accomplished through the utilization of a cascaded forward propagation–backpropagation neural network model [52,53,54]. The selection of a neural network for this purpose stemmed from the inherent complexity of auxetic structures, which involve a multitude of geometric parameters. The nonlinear relationship between these parameters and the Poisson’s ratio makes a neural network an apt choice for accurate prediction. In this approach, a regression model was effectively trained to predict the Poisson’s ratio based on the intricate geometric parameters characterizing the structure. The decision to employ a neural network was particularly justified by the intricate nature of the auxetic structure, where the large number of parameters contributed to the inherent nonlinearity in determining the Poisson’s ratio based on the geometric parameters.

**Figure 10:**
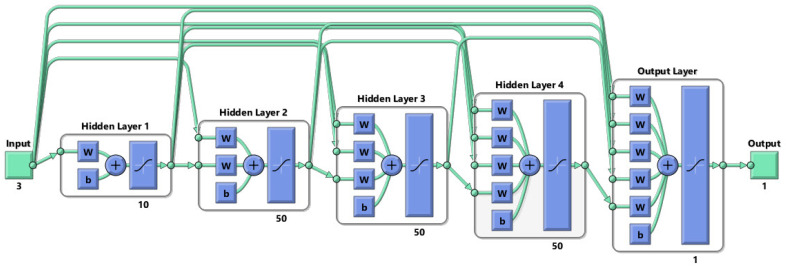
Schematic drawing for the cascaded neural network with 5 layers.

The process commenced with the forward propagation phase, where the model was supplied with input data containing the geometric parameters, including the side length (*a*), the side width (*b*), the overlap distance (*t*), the rotating angle between the units (θ), and the material properties of the auxetic structure. The computation of the weighted sums and the application of the activation functions were carried out by the model to generate predictions. These predictions were subsequently compared to the actual Poisson’s ratios present within the training data, enabling the model to compute the loss, which quantifies the disparity between the predicted and actual values. It is noteworthy that the model architecture comprised five layers, with the distribution of neurons across these layers as follows: 35 neurons in the initial layer, 45 neurons in the second layer, 55 neurons in the third layer, 45 neurons in the fourth layer, and 35 neurons in the final layer, as presented in Figure 10. This carefully configured architecture facilitated the intricate computations necessary for an accurate Poisson’s ratio prediction, especially crucial in the context of the complex and nonlinear relationship between the geometric parameters of auxetic structures and their Poisson’s ratios.

In addition to the determination of the Poisson’s ratio, the neural network training process was enhanced by the utilization of the “TrainLM” training function, which employs the Levenberg–Marquardt backpropagation algorithm. This widely utilized optimization algorithm combines the benefits of gradient descent and Gauss–Newton methods to efficiently minimize the network’s error or loss function. To further refine the training process, the “learnGDM” adaptation learning function incorporates the use of gradient descent with momentum (GDM). By incorporating a momentum term, this function enhances the convergence speed and stability of the network. The momentum term accumulates the effects of previous weight updates, aiding in the progress toward steeper gradients. In conjunction with these training functions, the TANSIG activation function is commonly employed to introduce nonlinearities and effectively model the intricate relationships between inputs and outputs. By mapping input values to a range between −1 and 1 using a sigmoid-shaped curve, the TANSIG function facilitates the accurate representation of the nonlinear relationships through its differentiability properties, enabling the use of backpropagation for weight and bias updates during the training phase.

**Figure 11:**
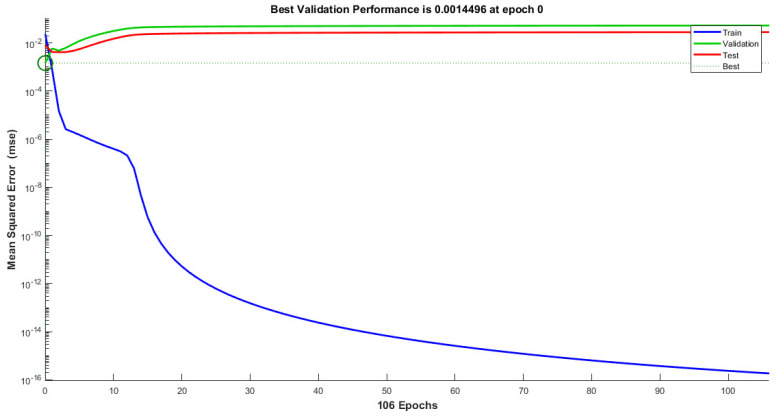
Neural network cascaded forward propagation–backpropagation performance analysis.

**Figure 12:**
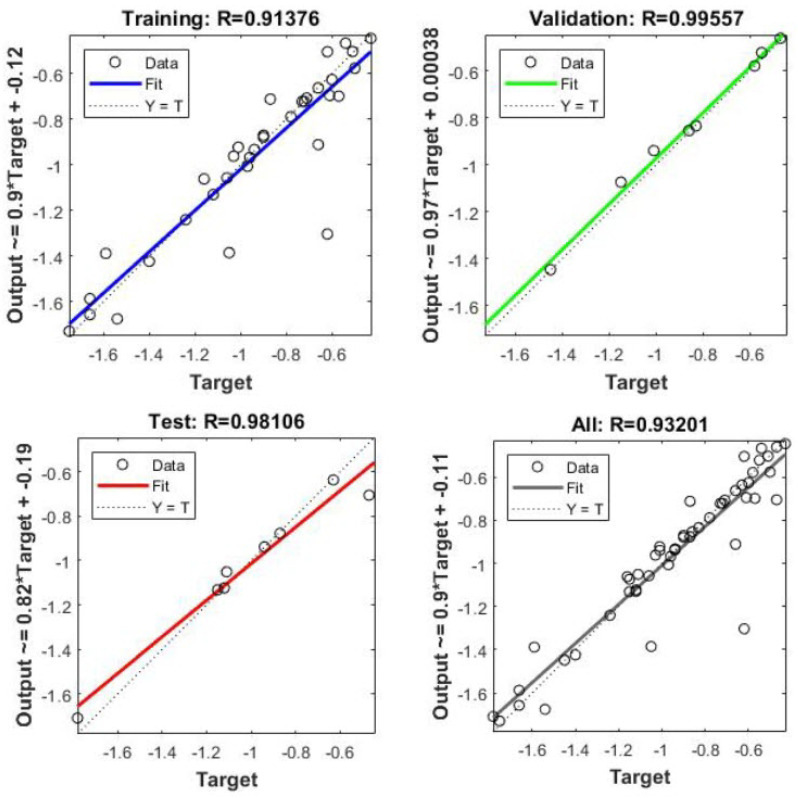
Training, validation, and testing of the neural network cascaded forward propagation–backpropagation for the auxetic model structure for predicting the Poisson’s ratio for different geometrical parameters.

Cascaded forward propagation–backpropagation offers several advantages in model regression for determining the auxetic structure’s Poisson’s ratio. Firstly, it allows the model to learn complex, nonlinear relationships between the input variables and output predictions. By propagating information through multiple layers, the model can capture intricate patterns and dependencies in the data, enabling more-accurate predictions. Additionally, the iterative nature of backpropagation enables the model to refine its parameters over time, gradually reducing the loss and improving the overall performance. This technique is widely used in the field of materials science and engineering, where accurate predictions of the material’s properties are crucial for designing and optimizing auxetic structures with specific Poisson’s ratios. The validation performance and regression curves are given in Figure 11 and Figure 12. To validate the performance of the artificial neural network (ANN) in predicting the negative Poisson’s ratio of auxetic structures, a comparison was conducted with FEA simulations. The maximum error observed in the predicted Poisson’s ratio was 8.62%, respectively. These results demonstrated the cascaded forward propagation–backpropagation neural network’s capability to accurately predict future values and validated its reliability and performance.

## 5. Conclusions

The present investigation involved a thorough examination of auxetic structures constructed from unit cells featuring rigid rotating squares or rectangles using finite-element analysis conducted through the ANSYS software. By manipulating various selected geometrical parameters, a diverse set of fifty geometries was generated, all of which exhibited auxetic behavior. Specifically, the angle (θ) between connected units, the side length (*a*), the side width (*b*) of the rotating rigid unit, and the overlap distance (*t*) used to connect the rotating rigid units within the structure were carefully chosen for manipulation. The comprehensive examination of different geometries and the accurate prediction of the Poisson’s ratio offered valuable insights for the design of auxetic structures using unit cells based on rotating squares and rectangles. The increase in the side width led to a significant rise in the values of the Poisson’s ratio, with a percentage increase ranging from 70.3% to 202.8%. These findings highlight the promising opportunities for the utilization of auxetic structures in various applications. The Poisson’s ratio for these auxetic structures was determined using a cascaded forward propagation–backpropagation neural network model. The model was trained to accurately predict the Poisson’s ratio based on the geometric parameters and material properties of the structure. The model architecture consisted of five layers with varying numbers of neurons, allowing for the accurate representation of complex relationships. The training process incorporated the “TrainLM” training function, which employed the Levenberg–Marquardt backpropagation algorithm, and the “learnGDM” adaptation learning function, which utilized gradient descent with momentum (GDM). The TANSIG activation function was employed to introduce nonlinearities and effectively model intricate dependencies. The validation of the neural network model was conducted by comparing its predictions with FEA simulations. The maximum error observed in the predicted Poisson’s ratio was 8.62%. This demonstrated the reliability and performance of the cascaded forward propagation–backpropagation neural network in accurately predicting the negative Poisson’s ratio of auxetic structures. The accurate prediction of the Poisson’s ratio by the neural network model enhanced the design process, enabling the creation of tailored structures with specific desired properties. The successful integration of finite-element analysis and neural network modeling contributes to the advancement of materials science and engineering, facilitating the optimization and development of innovative structures. 

## Figures and Tables

**Table 1:** Selected values for each parameter in the simulation study.

Geometrical Parameters	Unit	Values
Unit side length (*a*)	mm	10, 12.5, 15, 17.5, 20
Unit side width (*b*)	mm	10, 12.5, 15, 17.5, 20
Overlap distance (*t*)	mm	5, 7
Rotating angle between units (θ)	Degrees	30°

**Table 2:** Material properties for aluminum alloy AA6082T6.

Property	Value	Unit
Density	2.7	gm/cm^−3^
Elastic Modulus	7200	MPa
Tensile Strength, Ultimate	310	MPa
Tensile Strength, Yield	270	MPa
Poisson’s Ratio	0.33	
Thermal Conductivity	170	W/m-K

## Data Availability

Data are contained within the article.
